# Searching for a Putative Mechanism of RIZ2 Tumor-Promoting Function in Cancer Models

**DOI:** 10.3389/fonc.2020.583533

**Published:** 2021-01-29

**Authors:** Monica Rienzo, Anna Sorrentino, Erika Di Zazzo, Marzia Di Donato, Vincenzo Carafa, Maria Michela Marino, Caterina De Rosa, Patrizia Gazzerro, Gabriella Castoria, Lucia Altucci, Amelia Casamassimi, Ciro Abbondanza

**Affiliations:** ^1^ Department of Environmental, Biological, and Pharmaceutical Sciences and Technologies, University of Campania “Luigi Vanvitelli”, Caserta, Italy; ^2^ Department of Precision Medicine, University of Campania “Luigi Vanvitelli”, Naples, Italy; ^3^ Department of Medicine and Health Sciences “V. Tiberio”, University of Molise, Campobasso, Italy; ^4^ Department of Pharmacy, University of Salerno, Fisciano, Italy

**Keywords:** *PRDM2*, RIZ2 overexpression, microarray, cell proliferation, apoptosis, 3D models

## Abstract

Positive Regulatory Domain (PRDM) gene family members commonly express two main molecular variants, the PR-*plus* isoform usually acting as tumor suppressor and the PR-*minus* one functioning as oncogene. Accordingly, PRDM2/RIZ encodes for RIZ1 (PR-*plus*) and RIZ2 (PR-*minus*). In human cancers, genetic or epigenetic modifications induce RIZ1 silencing with an expression level imbalance in favor of RIZ2 that could be relevant for tumorigenesis. Additionally, in estrogen target cells and tissues, estradiol increases RIZ2 expression level with concurrent increase of cell proliferation and survival. Several attempts to study RIZ2 function in HeLa or MCF-7 cells by its over-expression were unsuccessful. Thus, we over-expressed RIZ2 in HEK-293 cells, which are both RIZ1 and RIZ2 positive but unresponsive to estrogens. The forced RIZ2 expression increased cell viability and growth, prompted the G2-to-M phase transition and organoids formation. Accordingly, microarray analysis revealed that RIZ2 regulates several genes involved in mitosis. Consistently, RIZ silencing in both estrogen-responsive MCF-7 and -unresponsive MDA-MB-231 cells induced a reduction of cell proliferation and an increase of apoptosis rate. Our findings add novel insights on the putative RIZ2 tumor-promoting functions, although additional attempts are warranted to depict the underlying molecular mechanism.

## Introduction

PR/SET Domain 2 (PRDM2) or Retinoblastoma Interacting Zinc finger (RIZ) protein is a member of the Positive Regulatory Domain (PRDM) gene family, which encodes for 19 different transcription factors in humans (1–3). All PRDM family members share an evolutionary conserved N-terminal domain, known as PR domain (PRDI-BF1-RIZ1 homologous), structurally and functionally similar to the SET domain (Su(var)3-9, Enhancer-of-zeste and Trithorax) (4–7). PR domain is followed by a variable number of Zinc finger domains towards the C-terminus, potentially mediating sequence-specific nucleic acid binding, protein-protein interactions or functioning in nuclear import (4–7). PRDMs are involved in epigenetic regulation of gene expression by acting as histone methyltransferases (HMTs) or by recruiting chromatin modifying enzymes (3, 5). Importantly, PRDM proteins function by tethering transcription factors to target gene promoters or by recognition of specific DNA consensus sequences *via* the Zinc-finger domains (6, 7). Of note, PRDM proteins contribute to many developmental processes, driving cell proliferation, differentiation, and maturation events by specifying cell fate choice or maintaining cell specialization through transduction of several cell signals (3, 4, 6).

Common feature of *PRDM* genes is the expression of two main molecular variants, generated by alternative splicing or alternative use of different promoters and differing for the presence or absence of the PR domain (3, 6, 7). The PRDM variants show an opposite, bivalent “yin-yang” behavior with the PR-*plus* isoform usually acting as a tumor suppressor, and the PR-*minus* one functioning as an oncogene (3, 7). Of note, the expression level imbalance between the two isoforms in favor of the PR-*minus* is often observed in many human malignancies being attributed to inactivating mutations or silencing of the complete form and/or increased expression of the PR-*minus* form (3, 6–8).

Specifically, *PRDM2* encodes for two main protein forms, known as RIZ1 or PRDM2a, containing the PR domain, and RIZ2 or PRDM2b lacking this domain (**Supplementary Figure S1**). RIZ2 transcript is generated by an internal TATA-less promoter localized at the 5**’** boundary of *PRDM2* coding exon 5 (3, 9). Both RIZ1 and RIZ2 are widely expressed in normal tissues in a similar ratio. However, the imbalance in their expression level could represent an important cause of malignancies (10, 11). Genetic evidences from tumor samples and cancer cell lines indicated that RIZ1 is a putative tumor suppressor gene commonly deleted (10), inactivated by point-mutations, especially those affecting its HMT activity (12–14), downregulated or silenced by DNA methylation of its promoter CpG island (13, 15–20). Altogether, these studies indicated that the PR domain through its HMT activity could play an important role in mediating RIZ1 tumor suppressor functions (14, 21). Furthermore, frameshift mutations of microsatellite repeats within the *PRDM2* coding region are frequently observed in colorectal, gastric, endometrial, and pancreatic cancers. Most of these mutations lead to a truncated protein at the C-terminal region containing the PR-binding motif, which is pivotal for RIZ1 folding, dimerization/oligomerization, and its PR domain-specific functions (12, 13, 22, 23). In addition, a microsatellite locus in *PRDM2* gene is frequently mutated in colorectal cancer implying its role as a driver mutation (24, 25). Of note, frameshift mutations in the *PRDM2* A (9) track have been observed in melanomas and nevi (26), as well as in leukemia cells (27).

PRDM2a/RIZ1 is also a component of the double-strand break (DSB) repair complex, which is essential for ensuring accurate repair outcome and genomic integrity maintenance (3, 8). Basically, RIZ1 cooperates with the macrohistone variant mH2A1.2 to direct the choice between the antagonistic DSB repair mediators, BRCA1 and 53BP1. The mH2A1/RIZ1 module enables a dynamic switch in chromatin conformation through H3K9me2 mediated by RIZ1. Then, a homologous recombination and repair through BRCA1 follows (28). Otherwise, the possible role of RIZ2 in this context has not been investigated so far.

Several studies have investigated the RIZ1 tumor suppressor functions. RIZ1 exerts growth inhibition and anti-cancer activities (29–33) and its ectopic expression induces cell growth arrest and apoptosis in a variety of cancer cells (15, 34, 35). In addition, RIZ1 mediates the estradiol proliferative effect in MCF-7 cell line, as a specific effector of estrogen action downstream of the hormone-receptor interaction. RIZ1, through its HMT activity, maintains gene-specific gatekeeper functions that prevent unliganded ER from binding to its target gene promoters and causing constitutive gene activation in the absence of stimulating signals. Upon estrogen-ER bound, RIZ1 become a coactivator able to induce the optimal estrogen response in female reproductive tissues (36, 37). Besides, RIZ1 silencing prompts cell proliferation (38, 39). Moreover, RIZ1 is expressed in normal prostate epithelial cells and is downregulated in cancer, with a switch of its sub-cellular localization from the nucleus to the cytoplasm upon cancer grade progression (40).

Likely, as a result of a positive selection related to RIZ2 promoting effects on proliferation, RIZ2 is always expressed in cancer cells (11, 15).

We have previously suggested that the Zinc-finger domains could be responsible for the oncogenic activity of RIZ2. MCF-7 cells expressing an Enhanced Green Fluorescent Protein (EGFP) fusion protein containing three of the eight putative Zinc-finger motifs common to both RIZ1 and RIZ2 showed a higher growth rate, being less sensitive to anti-estrogens growth inhibitory effect and expressing higher levels of cyclins D1 and A (41). We also demonstrated that RIZ2 is an ERα target gene, since an estrogen-responsive element (ERE) within the RIZ promoter 2 is regulated in a ligand-specific manner by ERα. Upon estradiol treatment a RIZ2 expression level increase was observed at both RNA and protein expression levels. The pattern of ERα binding, histone H4 acetylation, and histone H3 cyclical methylation of lysine 9 was comparable to other estrogen-regulated promoters. Association of topoisomerase IIβ with the RIZ promoter 2 confirmed the transcriptional activation induced by estrogens. This ligand-specific regulation induced the preferential transcription of exon 9a and a subsequent reduction in the amount of transcripts with exons 9b and 10, which determine different polyadenylation sites (11, 42).

The presence of transcripts with different UTRs suggests that a possible post-transcriptional control through miRNAs may occur. For instance, estradiol could modulate RIZ2/RIZ1 ratio also through various miRNAs that control estradiol response in breast cancer cells (43); interestingly, some of these target consensus sequences could be recognized in the exon 9 of PRDM2 gene by bioinformatics analysis (http://mirdb.org/cgi-bin/search.cgi). However, to date a mechanism involving PRDM2 regulation by these miRNAs can only be hypothesized. Furthermore, microarray analysis revealed PRDM2 as a target of the miR-17-92 cluster in human cholangiocarcinoma cells (44).

Despite several findings suggest that RIZ gene products are related to proliferation and apoptosis control with an opposite function, the molecular mechanisms and the involved cellular pathways through which RIZ2 displays oncogenic functions stay quite unclear.

Thus, the present study points to elucidate the putative mechanisms of the tumor-promoting function of RIZ2 isoform, through the investigation of its effects on regulated genes and enriched pathways by microarray analysis. Moreover, the biological consequences of RIZ2 overexpression in HEK-293 cells have been explored through some functional studies.

## Materials and Methods

### Cell Culture and Transfection

HEK-293, MCF-7, and MDA-MB-231 cells were grown and propagated as described elsewhere (11, 45).

MCF-7 and MDA-MB-231 cells were made quiescent as described (46), using phenol red-free Dulbecco’s modified Eagle Medium (DMEM) supplemented with 3% charcoal-dextran-stripped fetal bovine serum (FCS), 1 nM cortisol, and 10 ng/ml insulin.

HEK-293 were maintained at 37°C with 5% CO_2_ in humidified atmosphere in DMEM high glucose supplemented with 10% FCS, 1% Non-Essential Amino Acid (NEAA), and antibiotics (100 U/ml penicillin, 100 mg/ml streptomycin).

Cells were transfected with siRNAs and plasmid DNAs using Lipofectamine™ 2000 Reagent in OptiMem I Reduced Serum Medium (Life Technologies, Carlsbad, CA, USA) for 6 h, following the manufacturer’s instructions. After removal of reaction mixtures, MCF-7 and MDA-MB-231 cells were cultured for an additional 60 h in phenol red-free DMEM with 5% charcoal-dextran-stripped fetal bovine serum, 1 nM cortisol, 10 ng/ml insulin. For HEK-293 stable clones, selection was performed by addition of 0.8 mg/ml Geneticin G418 (Sigma-Aldrich). Positive colonies were selected by manual picking. At least two clones for each transfection were selected and characterized. Transfection was also verified by fluorescent microscopy analysis.

### RNA Interference (RNAi), Plasmids, and Constructs

The siRNA duplex (Dharmacon Research, Lafayette, CO, USA) designed for silencing of both RIZ gene products (siRNA-total) covered the region coding for aa 333–340 of RIZ1 protein or aa 132–139 of RIZ2 protein (sense 5’-GACUGCUCAGAGGUAACAC-3’). For each experiment, at least two concentrations of siRNA-total (35 and 50 nM) were transfected in the presence of an excess of tRNA (Ambion Inc., Austin, USA). An equal concentration of SilencerR Negative Control #1 siRNA (Ambion Inc.) was used as negative control. The efficiency of transfection was measured by labeling siRNA-total with equimolar amounts of fluorescein ULSR (Fermentas Inc., Hanover, MD, USA), according to manufacturer’s instruction, and expressed as the average of the percentage of fluorescent cells in each microscope field (five fields). Data derived from experiments with transfection efficiency greater than 55%. Experiments with differences >20% in transfection efficiency among different experimental points were discarded.

The pEGFP-C1 vector was purchased from Clontech (Palo Alto, CA, USA) and was used to clone in the BamH1 site, the sequence of RIZ2 open reading frame (NM_015866.4) (41). The primers used to amplify RIZ2 coding sequence with Bam H1 site restriction were as follows: RIZ2F (forward) (5’-AAGGATCCAGAGATTCTGCAGAATGGT-3’) and RIZ2R (reverse) (5’-AAGGATCCTACAGGAAGTTCCTGAAG-3’). A 4.42 kb RIZ2 fragment were recovered and ligated by T4 DNA Ligase (Promega, Madison, WI, USA). Recombinant plasmids were identified by restriction enzyme fragment analysis. In order to verify the correct orientation of the inserts and the integrity of the open reading frames, positive recombinant plasmids were directly sequenced with an ABI Prism Dye Terminator sequencing kit and analyzed on an ABI PRISM automated sequencer (Applied Biosystems) (47). The resultant plasmid expressing RIZ2, designated pEGFP_hRIZ2, was in frame with the EGFP coding sequence, with no intervening in-frame stop codons. Plasmids for transfection were prepared with Plasmid Midi Kit (Qiagen Inc, Valencia, CA, USA), according to manufacturer’s instructions. The EGFP expressed in the vectors was used to measure transfection efficiency (an average >80% of HEK-293 cells were transfected). For RIZ truncated C-terminal, the forward primer (5’-AAGGATCCCTGCAGACACCCTCCCTTT-3’) was employed instead of RIZ2F, which was utilized in transient transfection experiment.

### RNA Extraction, Quantitative and Semi-Quantitative Reverse Transcriptase Polymerase Chain Reaction (RT-PCR)

Total RNAs were extracted from cells using Trizol solution (Thermofisher), according to the manufacturer’s instructions. RNA samples were then treated with RNase-free DNase-I (Boehringer Mannheim, Indianapolis, IN, USA). The integrity and quantity of RNAs were assessed by denaturing agarose gel electrophoresis and by spectrophotometry analysis. Then, 500 ng total RNA was reverse transcribed with SuperScript III (Thermofisher); 1 μl of the reverse-transcriptase reaction was used as a template in a PCR reaction as previously described (39). The amplification products were also analyzed by agarose gel electrophoresis. *GAPDH* was used as housekeeping control gene. Quantitative RT-PCR analysis was performed using the SYBR Green PCR Master Mix (Applied Biosystems, Foster City, CA, USA), 160 nM of each primer and about 50ng of cDNA (RNA equivalent) as template in an iCycler thermocycler (Bio-Rad Laboratories Inc., Hercules, CA, USA). PCR condition were 95°C for 4 min followed by 51 cycles of 20 s at 95°C, 45 s at 60°C, and 45 s at 70°C. RIZ1 transcript amplification was performed with the following primers: 118F (5’-CTG GAT CCA CCC GGA TTG GTG TCT GGG-3’) and 438R (5’- TCG GAT CCA GGG TTG TCT TCC CCA TTG TAC C-3’). Primers 649F (5’-CTG GAT CCT CAG CCT CAG CAC TTG AGC AG-3’) and 975R (5’-TCG GAT CCT GTT TTT GGT TCC TCT AAT AAA TCT TC-3’) were used for analysis of all RIZ transcripts (34, 39). All reactions were carried out at least in triplicate for every cDNA template and the melting curves were analyzed to verify the specificity of reaction. The relative gene expression was calculated using the 2^ΔΔCt^ method (48). *GAPDH* was used as a housekeeping gene for normalization. Differences between two experimental groups were analyzed by the Student’s t-test. Differences between means were considered significant at P < 0.05 (47, 49).

### Western Blot Assay and Densitometric Analysis

Electrophoresis and Western blot analysis were performed as described elsewhere (38) with mouse monoclonal antibodies to green fluorescent protein (GFP) from Roche (Roche, Mannheim, Germany) and mouse monoclonal antibody RZ2413 (1 mg/ml) to a synthetic peptide at residues 960–972 containing RIZ nuclear receptor box protein (41).

Western blot analysis of total cell extract of interfered MCF-7 and MDA-MB-231 were revealed with commercial polyclonal antibodies to RIZ1 protein (aa 6-22) or with commercial antibodies to human Cyclin B1, and histone H1.1 (Abcam Ltd., Cambridge, UK).

### Immunofluorescence and Cytoskeleton Analysis

HEK 293-pEGFP and HEK 293-pEGFP_hRIZ2 were plated on gelatin-coated coverslips. After 24 h, cells on coverslips were fixed in 4% paraformaldehyde and permeabilized using diluted (0,2% in PBS) Triton-X100 at room temperature. Nuclei were stained with 1 μg/ml Hoechst 33258 (Sigma). Cytoskeleton analysis was performed by Texas red-labeled phalloidin (Sigma-Aldrich), as reported elsewhere (50). Fields were analyzed with a DMBL Leica fluorescence microscope equipped with HCX PL Fluotar 100 × oil objective. Representative images from three independent experiments were captured using a DC480 camera (Leica) and acquired by Application Suite (Leica) software (50).

### Cell Cycle Analysis

HEK 293-pEGFP and HEK 293-pEGFP_hRIZ2 cells (2 × 10^5^ cells/well) were seeded into six-well plates. After 24 h, cells were harvested, centrifuged at 1,200 rpm for 5 min, and resuspended in 500 μl of cell cycle buffer solution (0.1% sodium citrate, 0.1% NP-40, RNase A, and 50 mg/ml Propidium Iodide -PI in PBS 1X). The results were acquired on fluorescence-activated cell sorting- FACS Calibur (BD Biosciences). Each experiment was performed in biological triplicates and values expressed as mean ± standard deviation. Mitotic blockade was obtained through cell treatment with 100 ng/ml nocodazole for 18 h (Sigma-Aldrich Co.). Cell distribution in the G1, S, and G2/M phases of the cell cycle was calculated from the resulting DNA histogram with BD CellQuest software.

Cell cycle distribution of PI-labeled MCF-7 and MDA-MB-231 cells was obtained by FACS analyses at 40 h from transfection with reported concentrations of siRNA-total.

### Clonogenic Assay

HEK 293-pEGFP and HEK 293-pEGFP_hRIZ2 cells (3 × 10^2^) were seeded into six-well plates and cultured at 37°C for ~10 days until cells have formed sufficiently large clones (at least 50 cells). Fresh media were supplied every 3 days. Clones were counted after 30 min fixing with a mixture of 6% glutaraldehyde and 0.5% crystal violet (51). The stained colonies were photographed and the number colonies with sizes ≥1 mm were counted using the ImageJ software (National Institutes of Health, USA) and expressed as mean ± S.E.M. Each assay was performed in at least three independent experiments in triplicate.

### HEK 293 Cell Viability

HEK 293-pEGFP and HEK 293-pEGFP_hRIZ2 cells (5 × 10^3^ cells/well) were seeded into 96-well plates. Cell viability was assessed by the 3-(4,5-dimethylthiazol-2-yl)-2,5-biphenyltetrazolium bromide (MTT) assay (Sigma-Aldrich Co.) at 0, 24, 48, and 72 h, as previously described (52). Absorbance was measured at 570 nm wavelength and at 690 nm for background subtraction. To estimate cell number through absorbance of solution, the regression lines of OD570-690nm values on serial dilutions of HEK293 cells, both pEGFP_RIZ2 and control cells was generated (53).

### MCF-7 and MDA-MB-231 Cell Number

MCF-7 and MDA-MB-231 cells were counted by the method of optical microscopy in the Bürker chamber. Cells interfered with two different siRNA concentrations (35 and 50 nM) were compared with untreated cells.

### Miniaturized 3D Cultures in Extracellular Matrix (ECM)

Miniaturized 3D cultures in Matrigel were performed as reported (54). Briefly, cell suspension containing 3 × 10^4^ cells was mixed with 200 µl of Matrigel Growth Factor Reduced (GFR) Basement Membrane Matrix (BD Biosciences) for each well and the embedding method was used to establish organoids (55). The mixture was seeded in 24-well plate and allowed to solidify for 45 min at 37°C, before the addition of 400 µl organoid plating medium to each well. Organoid plating medium was made using DMEM/F12 medium, containing 10% FBS, penicillin (100 U/ml), streptomycin (100 U/ml), diluted GlutaMAX 100X, 10 mM Hepes, 1M nicotinamide, 500 mM N-acetylcysteine, and 10 µM Y-27632 (Millipore, Burlington, MA, USA). After 3 days, when the organoid structure was visible, the organoid-plating medium was replaced with a similar medium without N-acetylcysteine and Y-27632. The medium was changed every 3 days. Different fields were analyzed using DMIRB Leica (Leica) microscope equipped with HCL PX Fluotar 40× and 63× objectives (Leica). At the indicated times, phase-contrast and immunofluorescence microscopy images were acquired using a DFC 450C camera (Leica). Images are representative of three independent experiments, each performed in duplicate. The relative organoid size (area) was calculated using the Application Suite Software and expressed as a fold increase over the organoid area calculated at 3th day.

### Human Gene Expression Microarrays

Agilent Technologies SurePrint Gene Expression Microarrays (Agilent Techologies, Santa Clara, CA, USA) were used to profile gene expression of RNA samples. Samples were processed according to Agilent Two-color Microarray Based Gene Expression Analysis (Low Input Quick Amp Labeling Kit protocol) by Agilent Spike-In Kit, according to the manufacturer’s instructions. The Agilent Two-Color Microarray-based Gene Expression Analysis uses Cyanine 3- and Cyanine 5-labeled targets to measure gene expression in experimental and control samples. Equal amounts of Cyanine 3-labeled (control samples) and Cyanine 5-labeled (experimental samples) cRNA from samples were simultaneously co-hybridized onto the arrayed oligonucleotides on the same Agilent 44k Whole Human Genome chip (Agilent Technologies, 4 × 44k format) slide at 65°C for 17 h using an Agilent Gene Expression Hybridization Kit in Agilent’s SureHyb Hybridization Chambers (G2545A). The hybridized microarrays were washed according to manufacturer’s instructions, and then scanned by Agilent Feature Extraction software (10.5, Protocol GE2_105_Dec08).

### Array Data Analysis

Data were analyzed *via* the R packages Limma and Hdaarray, both available in R. Specifically, Limma is an R package for the analysis of gene expression microarray data, especially the use of linear models for analyzing designed experiments and the assessment of differential expression (56). Limma allows to analyze comparisons between many RNA targets simultaneously in arbitrary complicated designed experiments. Empirical Bayesian methods are used to provide stable results even when the number of arrays is small. Expression intensities were at first background corrected. Then, they were normalized so that the intensities or log-ratios have similar distributions across a set of arrays. A regularized version of the T-test was then applied (57). A FRD correction for multiplicity was applied.

### GO Functional and Pathway Analyses

GO analysis is a commonly applied method for the functional annotation of large-scale expression data. The KEGG pathways database is a comprehensive and recognized database with a wide range of biochemical pathways, linking genomic information with higher-order functional information. Gene ontology and pathway analysis were carried out using Database for Annotation, Visualization, and Integrated Discovery (DAVID) v.6.7 and Reactome [(58–60); https://david.ncifcrf.gov/; https://reactome.org/]. The list of DEGs with a more stringent level of P < 0.01 was used to limit the input to DAVID to achieve meaningful overrepresented data. From the obtained output, GO FAT terms were used instead of GO ALL, because the FAT category filters out the very broad GO terms based on a measured specificity of each term to yield more specific terms. Using these data, differences in biological processes (BP), molecular function (MF), cellular component (CC) were detected. Significant enrichment was considered when enriched gene count ≥2, and p value <0.05. Pathways with a P < 0.05 were considered.

### Statistical Analysis

Results are reported as mean ± Standard Deviation (SD). Three independent experiments in triplicates (n ≥ 9) were performed. Differences between experimental groups were analyzed by the Student’s t-test. All statistical analyses have been performed using JMP Software purchased by Statistical Discovery SAS Institute. Finally, differences between means were considered significant at *p < 0.05 and particularly significant at **p < 0.01.

## Results

### RIZ2 Overexpression in HEK-293 Cell Line

**Figure 1:**
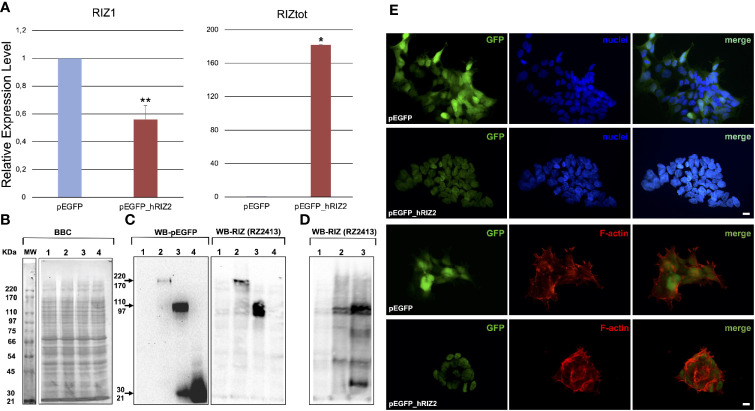
*RIZ2 overexpression in HEK-293 cell line.* **(A)** Histograms represent the relative expression level of RIZ1 and RIZtot by qRT-PCR analysis using two sets of primers: 118F/438R recognizing sequences on exons 3 and 5 (exclusive of RIZ1) and 649F/975R recognizing a C-terminal region common to both RIZ1 and RIZ2 and indicated as RIZtot. The relative expression level is indicated as fold changes from HEK 293-pEGFP cells. Data were obtained from three independent experiments and expressed as mean ± SD. **p < 0.01 and *p < 0.0005 for RIZ1 or RIZtot *versus* control. **(B)** A representative Coomassie brilliant blue (BBC) stained gel. **(C, D)** Representative western blot analyses of total cell lysates from HEK-293 cells transiently **(C)** or stable **(D)** transfected with pEGFP or pEGFP_hRIZ2. Antibodies against RIZ (RZ2413) and EGFP were used in western blotting analysis. Sample 1, empty vector; sample 2, HEK 293-pEGFP_hRIZ2; sample 3, pEGFP-hRIZaa923-COOH; sample 4, pEGFP). **(E)** Immunofluorescence analysis of GFP (left panel) and F-actin (lower section, middle panel) in HEK 293-pEGFP and HEK 293-pEGFP_hRIZ2. Nuclei are stained in blue (upper section, middle panel). Merged images are shown in the right panels. The images shown are representative of three independent experiments. Images were captured using a DC480 camera (Leica) and acquired by Application Suite (Leica) software.

In order to investigate the RIZ2 oncogenic mechanisms of action together with the pathways impacted, RIZ2 was overexpressed in the normal human embryonic kidney HEK-293 cell line, both RIZ1 and RIZ2 positive and unresponsive to estrogens. To this purpose, we transfected HEK-293 cells with a plasmid encoding for RIZ2 in frame with EGFP (pEGFP_hRIZ2) and with the E-GFP empty vector (pEGFP). In this way, the balance between RIZ1 and RIZ2 was modified in favor to RIZ2, reproducing a condition often observed in cancer (3, 8). Quantitative assay of the RIZ2 transcript was unfeasible because of the extensive similarity between the two gene products, RIZ1 and RIZ2. The RIZ2 overexpression was verified after transfection by qRT-PCR of reverse-transcribed total cellular RNA, using two sets of primers: 118F/438R and 649F/975R recognizing sequences on exons 3 and 5 (exclusive of RIZ1) or on exon 8 (common to both RIZ1 and RIZ2 and indicated as RIZtot) respectively in both transient (data not shown) and stable transfected cells (**Figure S1** and **Figure 1A**) (34, 39). qRT-PCR analysis revealed a highly significant increase of RIZ2 expression levels with a decrease in RIZ1 expression levels compared to control cells suggesting a putative mechanism of transcription autoregulation. RIZ2 protein expression level was evaluated by Western blot analysis with the monoclonal antibody RZ2413 (41) recognizing the residues 960–972 of the proline rich domain (aa sequence 952–1,052) common to RIZ1 and RIZ2 forms in transient (**Figures 1B, C**) and stable transfected (**Figure 1D**) cells. A RIZ2 overexpression was revealed in transiently transfected cells (**Figure 1C**). Western blot analysis of RIZ gene products in pEGFP-hRIZ2 stable transfected cells, revealed a band of 162kDa corresponding to the predictable MW from the primary sequence of RIZ2 (http://www.ensembl.org/index.html) that is weaker than observed in transient transfection (**Figure 1D**). Additionally, the WB analysis showed specific immunoreactive bands migrating at 110–90 kDa, likely resulting from processing events, as previously reported (11) (**Figures 1C, D**). Altogether, both qRT-PCR and Western blot analysis confirmed RIZ2 overexpression. As expected, fluorescence microscopy analysis revealed that RIZ2 was predominantly localized in the nucleus (**Figure 1E**). Furthermore, HEK-293 cells overexpressing RIZ2 showed an increased content of thickened F-actin on the periphery of the cells with pronounced membrane protrusions, such as filopodia and lamellipodia (**Figure 1E**). These findings indicate that RIZ2 overexpression induces cytoskeleton changes.

### Gene Ontology and Pathway Enrichment Analyses

In order to evaluate the effects of RIZ2 overexpression on gene expression, a pilot expression study was performed through microarrays analysis. We compared the differential gene expression between HEK-293 cells overexpressing RIZ2 (pEGFP_hRIZ2) *versus* control cells transfected with the E-GFP empty vector (pEGFP). The obtained data have been deposited in NCBI’s Gene Expression Omnibus (61) and are accessible through GEO Series accession number GSE150031 (https://www.ncbi.nlm.nih.gov/geo/query/acc.cgi?acc=GSE150031). Differentially expressed genes (DEGs) were initially selected on the basis of an adjusted P-value <0.05 (2520 DEGs). Given the huge number of DEGs obtained, a more stringent level of *P* < 0.01 was applied to generate the list of differentially expressed probe sets used for functional categorization through gene ontology (GO) overrepresentation analysis (595 DEGs). A total of 595 DEGs were identified in pEGFP-hRIZ2 cells compared to control cells, including 292 downregulated (49.1%) and 303 upregulated (50.9%).

**Figure 2:**
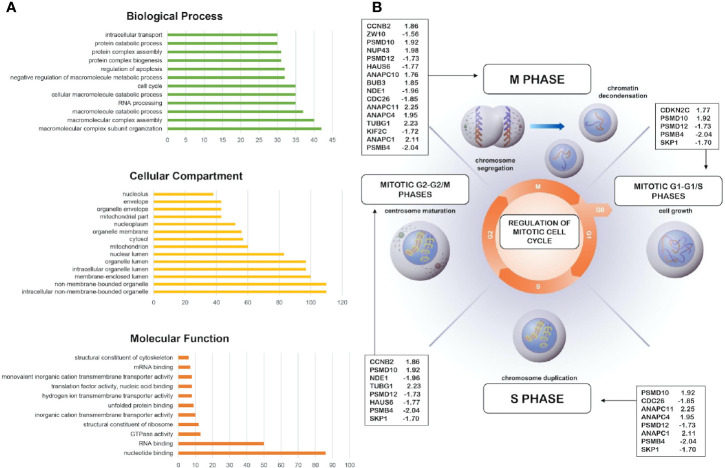
*Bioinformatics analysis of the differentially expressed genes and pathway analysis.* **(A)** More representative Gene Ontology enrichment of differentially expressed genes (DEGs) in cellular component, molecular function, and biological process. Count: number of genes related to the enriched GO. **(B)** Overrepresentation and enrichment analysis of DEGs in cell cycle, mitotic phase (R-HSA-69278). The fold change value of indicated genes are reported.

The GO analysis was performed through DAVID online tool and allowed classifying DEGs into three categories, including the biological process, the cellular component, and the molecular function (**Figure 2A**; **Tables S1–S3**) (https://david.ncifcrf.gov/). As shown in **Table S1**, in the biological process category DEGs were enriched in different GO function such as in macromolecular complex assembly, cell cycle, mRNA processing, RNA splicing, macromolecule catabolic process, intracellular transport, RNA processing (**Table S1**). In the cellular component category, the identified DEGs were significantly associated with intracellular organelle lumen, non-membrane-bounded organelle, membrane-enclosed lumen, organelle lumen, nuclear lumen, mitochondrion, cytosol, nucleoplasm, and organelle membrane (**Table S2**). For the molecular function category, enrichment of DEGs was revealed in RNA binding, structural molecule activity, transcription cofactor activity, nucleotide binding (**Table S3**).

DEGs functional and signaling pathway enrichment was performed using online websites of KEGG pathway in DAVID online tool (https://david.ncifcrf.gov/). The upregulated genes mainly enriched in oxidative phosphorylation (p = 5.9E-4) and cell cycle (p = 2.3E-3) whereas the downregulated genes mainly enriched in spliceosome signaling pathways (p = 1.1E-2) (**Table S4**). Additionally, DEGs were analyzed using the “Reactome” website in order to study and visualize pathway overrepresentation (enrichment) and representation of expression data viewed as an overlay on “reactome” pathways (https://reactome.org/). In this case, pathway analysis using this database, revealed cell cycle, metabolism, gene expression (transcription), and protein localization as significantly enriched pathways. In particular, several DEGs (i.e. *CCNB2*, *CDKNC2*, *CDC26*, *PSMD10*) were overrepresented in cell cycle, mitotic phase (R-HSA-69278) hypothesizing a possible role of RIZ2 during cell cycle progression (**Figure 2B**).

**Supplementary Figure S2** shows the top SP-PIR keywords of enriched DEGs. Many DEGs were associated with phosphoprotein (46.1%; p = 2.1E-9), alternative splicing (40.7%; p = 5.7E-3), nucleus (29.8%; p = 4.3E-8), acetylation (26.4%; p = 5.5E-20, and cytoplasm (20.2%; p = 3.2E-3) (**Table S4**).

### Effects of RIZ2 Overexpression on HEK-293 Cell Viability and Cell Cycle Progression

**Figure 3:**
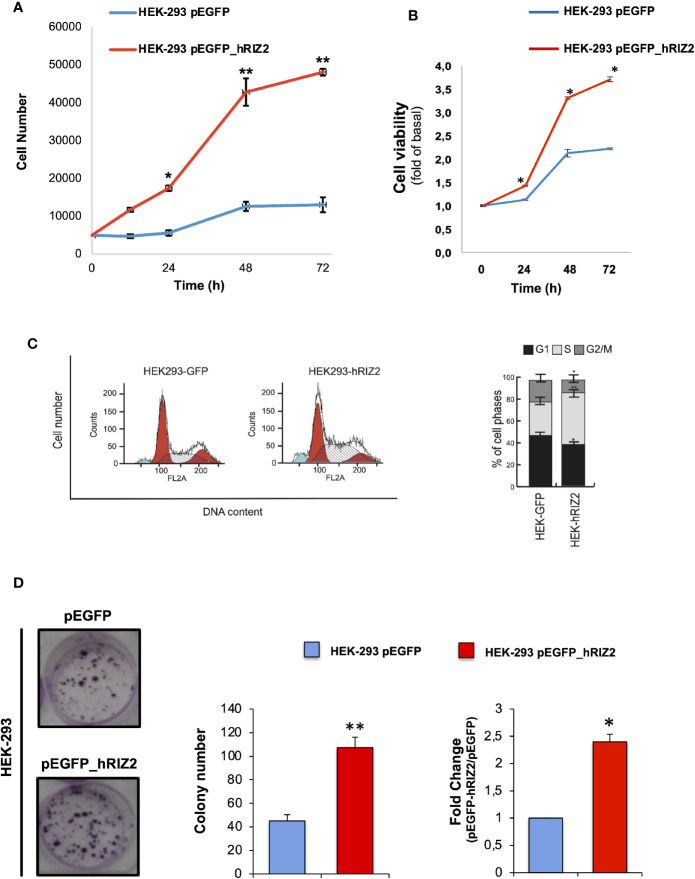
*Effects of RIZ2 overexpression on HEK-293 cell Viability and cell cycle progression* **(A, B)** Effect of RIZ2 overexpression on HEK-293 cell viability by MTT assay. **(A)** HEK-293 cells stable transfected with pEGFP_hRIZ2 or with pEGFP alone, as control, were cultured for 24, 48, and 72 h. Graph represents the extrapolated cell number at the different time points. **(B)** HEK-293 cells stable transfected with pEGFP_hRIZ2 or with pEGFP alone, as control, were cultured for 24, 48, and 72 h. Graph represents the cell viability, expressed as fold increase over the basal. Three independent experiments were done. Means and standard error of the means (SEMs) are shown. *p < 0.005 for the indicated experimental points *vs.* the corresponding untreated control. **(C)** Effects of pEGFP_hRIZ2 overexpression on HEK293 cell cycle regulation. Histogram Plots of cell cycle distribution (left) and cell cycle analysis (right) are illustrated. Graphs show the mean of at least three independent experiments with error bars indicating standard deviation. Values are mean ± standard deviation (SD) of biological triplicates. ****p-value ≤ 0.0001, ***p-value ≤ 0.001, **p-value ≤ 0.01, *p-value ≤ 0.05, ns p-value > 0.05 *vs.* pEGFP control cells. **(D)** Representative image of clonogenic assay. Ten days after seeding, clones were counted, and their cellularity was evaluated by phase contrast microscopy. The histogram represents the average number of colonies of at least three independent experiments, each performed in triplicate (**P < 0.0001 *vs.* empty vector); on the right the fold change in colony number formation between pEGFP_hRIZ2 cells and pEGFP control cells (*P < 0.001 *vs.* empty vector).

In order to investigate the role of RIZ2 in the control of cell viability and survival, colorimetric MTT assay was performed in HEK-293 cells stable transfected with pEGFP_hRIZ2 or with pEGFP control vector after 24, 48, and 72 h. The number of HEK 293-pEGFP_hRIZ2 cells at 24, 48, and 72 h was greater than HEK 293-pEGFP (**Figure 3A**). As shown in **Figure 3B**, RIZ2 overexpressing cells exhibited a significant increase in cell viability, as compared with the control cells at 48 and 72 h.

The biological effect of RIZ2 overexpression on cell cycle progression was evaluated through FACS analysis using HEK-293 stable overexpressing pEGFP_hRIZ2 or pEGFP vectors. pEGFP_hRIZ2 clones showed a decrease of cell percentages in G1 and G2/M phases (G1, 38,79 *vs* 47,73%; G2/M 12,10 *vs* 20,54%) as well as an increase of cell percentages in S phase (49,12 *vs* 31,73%; **Figure 3C**), as compared to the control cells. To better discriminate the observed effects, cells were synchronized with nocodazole treatment for 16 h (**Supplementary Figure S3**). Three hours after nocodazole release, cells were recovered, and cell cycle analysis was done. After synchronization, FACS analysis confirmed the decrease of cells in G2 phase (25,52 *vs* 34,45%) and the increase of cells in S phase (44,21 *vs* 31,20%). No significant effects were observed in G1 phase. Altogether, these data indicate that RIZ2 could promote the G2-to-M phase transition.

To deeper assess the role of RIZ2 in tumorigenesis, we also analyzed the ability of HEK-293 cells stable overexpressing pEGFP_hRIZ2 or the pEGFP control vector to form colonies. To this purpose, HEK-293 stable transfected cells were seeded at low density in six-well plates. Ten days later colonies were counted, and their cellularity was evaluated by phase-contrast microscopy. Overexpression of RIZ2 induced the formation of a markedly higher number of colonies than the control cells (**P < 0.0001 *versus* empty vector; *P < 0.001 *versus* empty vector) (**Figure 3D**).

In summary, these data suggest that RIZ2 may exert a promoting role in cell cycle progression and tumor formation.

### RIZ2 Overexpression Increases HEK-293 3D-Organoid Growth

3D cell culture systems are mini organ-like structures capable of self-renewal and self-organization, which closely recapitulate the *in vivo* microenvironment as well as the molecular and genetic signature of tissues or organs of origin (62).

**Figure 4:**
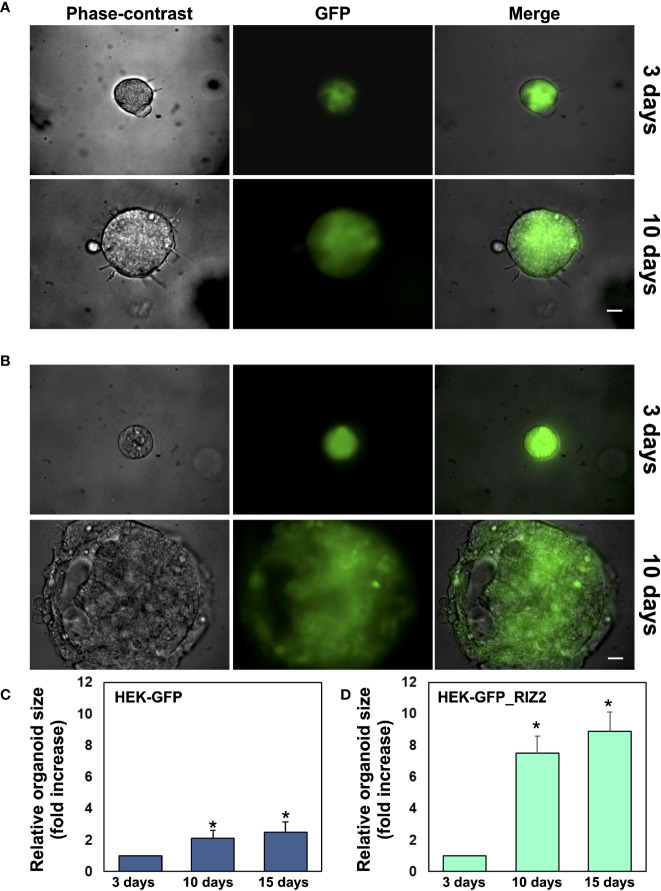
*RIZ2 overexpression Increases HEK-293 3D-Organoid Growth.* In **(A)** HEK-293 cells stable transfected with pEGFP were used in miniaturized 3D cultures in extracellular matrix (ECM). In **(B)** HEK-293 cells stable transfected with pEGFP_hRIZ2 were used in miniaturized 3D cultures in ECM. In **(A, B)**, at the indicated times, phase-contrast- (left panels), immunofluorescence- (middle panels), and merged (right panels) microscopy images were captured using DMIRB Leica (Leica) microscope equipped with C-Plan 40× objective (Leica) and acquired using a DFC 450C camera (Leica). Scale bar, 100 µ. Images are representative of three independent experiments, each performed in duplicate. **(C, D)** Graphs represent the relative organoid size (area), which was calculated using the Application Suite Software and expressed as a relative fold increase over the organoid area calculated at 3^th^ day. Means and standard error of the means (SEMs) are shown. *p < 0.05 for the indicated pEGFP_hRIZ2 HEK-293 cells *vs.* the corresponding pEGFP HEK-293 control cells.

To develop cell culture models resuming cancer tissues and to reproduce the complex *in vivo* architecture, 3D organoids were established. Phase-contrast and immunofluorescence images at 3 days revealed a 3D structure in both pEGFP (**Figure 4A**) and pEGFP overexpressing RIZ2 HEK-293 cells (**Figure 4B**) cultured in Matrigel. Both cell types generated roundish and well-differentiated organoids. Changes in dimension and structure of organoids were then monitored for additional 15 days. Phase-contrast and immunofluorescence microscopy images at 10^th^ day were captured and shown (**Figures 4A, B**). Quantification of data was also done, and the graphs in **Figures 4C, D** show that pEGFP organoid size was increased by about 2- and 2,5-fold after 10 and 15 days, respectively. Interestingly, we observed a significant (p < 0.05) increase (about 7,5- and 9-fold at 10 and 15 days, respectively) in the size of pEGFP hRIZ2-derived organoids (**Figures 4C, D**).

In conclusion, these findings demonstrate for the first time a role for RIZ2 in the growth of HEK293-derived organoids.

### RIZ Silencing in MCF-7 and MDA-MB-231 Cell Lines

Based on our findings on the dual role of PRDM2 products in the control of cell proliferation and survival in MCF-7 cell line (39), we aimed at clarifying whether the effects previously observed in MCF-7 cells are merely due to RIZ1 silencing or the resulting imbalance in favor of RIZ2.

**Figure 5:**
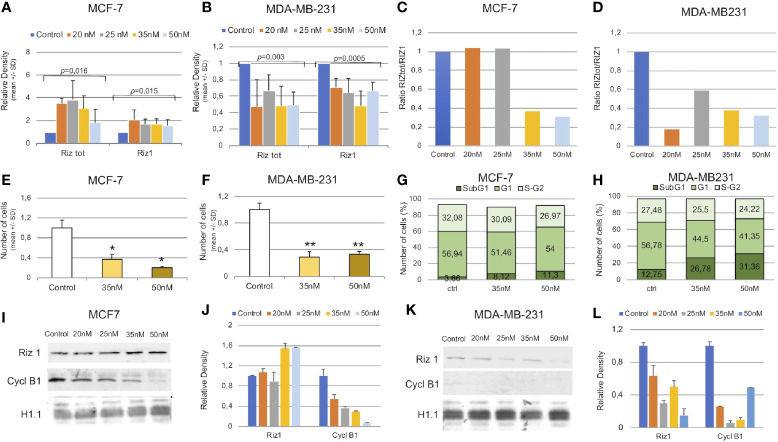
*RIZ2 silencing in MCF-7 and MDA-MB-231 cell line.* **(A, B)** Relative expression by semi-quantitative RT-PCR of RIZtot transcripts in cells interfered with different concentrations of siRNA-total (20, 25, 35, and 50 nM). **(C, D)** Histograms representing the variation of RIZ2/RIZ1 ratio are shown. **(E, F)** Cell counts in cells interfered with two different concentrations (35 and 50 nM) compared with untreated cells (*p < 0.05 and **p < 0.01). **(G, H)** Cell cycle distribution of PI-labeled cells by FACS analyses at 40 h from transfection with reported concentrations of siRNA-total. **(I–L)** Representative Western blot analysis of total interfered cell extracts with the indicated antibodies and the relative densitometry analysis.

To this purpose, a RIZtot mRNA interference experiment was set up in MCF-7 and MDA-MB-231 breast cancer cell lines with a siRNA spanning a sequence coding for a region common to both RIZ1 and RIZ2 gene products (aa 333–340 of RIZ1 protein or aa 132–139 of RIZ2 protein), indicated as siRNA-total (see also **Figure S1**). The siRNA was designed taking into account the principles able to ensure efficiency and specificity of target gene knockdown (63–65). Specifically, the selected siRNA-total sequence is near to the ATG initiation codon for the RIZ2 protein and not able to efficaciously interfere the RIZ1 product, whose ATG start codon is localized at about 1,000 nucleotides upstream (65). Thus, siRNA-total interference resulted much more efficient towards RIZ2 than RIZ1 transcripts. Different concentrations of siRNA-total (20, 25, 35, and 50 nM) were initially used. MCF-7 and MDA-MB-231 cells are ERα- positive and negative respectively, with the first expressing both RIZ1 and RIZ2, while the latter expressing very low levels of RIZ1 but substantial levels of RIZ2 (11) (**Figures 5A, B**, and **Supplementary Figure S4**). Transfection of MCF-7 cells with the various concentrations of siRNA-total produced an increase in the amounts of total transcripts, as evidenced from the analysis of the RT-PCR amplified fragments with primers complementary to sequences common to RIZ1 and RIZ2, including exon 8 (**Figure 5A**). The analysis of the same RNAs with primers complementary to a RIZ1-specific sequence including exons 3, 4, and 5, showed also an increase of the RIZ1-specific amplicon in the cells treated with the different concentrations of siRNA-total (**Figure 5A**). These findings suggest that RIZ1 transcript is upregulated in RIZtot MCF-7 interfered cells. Thus, this silencing modified the balance between RIZ1 and RIZ2 in favor to RIZ1, suggesting that the ratio between RIZ1 and RIZ2 is pivotal and the two molecular variants regulate each other (**Figure 5C**). Besides, transfection of MDA-MB-231 cells, expressing very low level of RIZ1, with increasing concentrations of the same siRNAs produced a decrease in the amount of total RIZ products and a concomitant decrease in RIZ1 expression level (**Figures 5B, D**).

We next analyzed the effect of the imbalance of RIZ2/RIZ1 ratio on cell viability. For this purpose, we used 35 and 50 nM of siRNA-total to silence RIZ2 in both cell lines. RIZtot silencing produced a significant decrease of MCF-7 and MDA-MB-231 cell numbers, as compared to control cells (**Figures 5E, F**). Additionally, cell cycle analysis indicated an increase of SubG1 phase in interfered cells over the control, suggesting an increase of the cells undergoing apoptosis especially when we used siRNA-total 50 nM; besides, a reduction of S-G2 was detected (**Figures 5G, H**).

We then analyzed also the protein levels of RIZ1 (**Figures 5I–L**). Results observed by RT-PCR analysis were confirmed by western blot technique. In MCF-7 cells, a sharp increase in the RIZ1 expression level was observed at the different concentrations of siRNA used (**Figures 5I–L**). In MDA-MB-231, instead, RIZtot silencing reduced the RIZ1 expression level. In this context, a reduction of cyclin B1 expression level was shown in both siRNA transfected cell lines (**Figures 5I–L**).

## Discussion

PRDM2, a member of the PRDM family, is involved in the proliferation and apoptosis control and acts in the transcriptional regulation of genes encoding for proteins implicated in development, cell cycle progression and cell adhesion. Several evidences have suggested that an imbalance in the expression levels of *PRDM2* main proteins, RIZ1 and RIZ2, could be relevant for neoplastic transformation (3, 8, 10). However, the underlying molecular mechanisms and the related cellular pathways are still unclear. RIZ1 functions have been extensively investigated (21, 36). In many reports, genetic evidence from tumor samples and cancer cell lines indicates that RIZ1 is a putative tumor suppressor gene (3). In addition, its ectopic expression is able to induce cell growth blockade and apoptosis in a variety of cancer cell lines (15, 33). Genetic or epigenetic modifications of the PRDM2/RIZ gene observed in human cancers lead to silencing of RIZ1 expression (12, 16, 22, 66), while, in contrast, they do not affect RIZ2 expression level. These findings suggest a positive selection for RIZ2 expression that could be related to its promotion of cell proliferation (15). Over the past decades, few studies have focused on RIZ2 biological activity. The main experimental pitfalls are related to the sequence identity of RIZ2 and RIZ1. Firstly, RT-PCR analysis is not able to evaluate the RIZ2 expression level but only the sum of RIZ1 and RIZ2 expression levels. Finally, the commonly used and commercially available antibodies raised against the common C-terminal domain do not enable the unique identification of RIZ2. As such, the inability to specifically detect RIZ2 has obviously hindered the study of its functional roles. In addition, in accordance with previously published experiments, pEGFP_hRIZ2 stable transfected HEK-293 cells showed a weaker RIZ2 specific product expression, than observed in transient transfection, accompanied by processed proteins of ≅110–90 kDa, which have a similar MW to the main product of a plasmid construct with RIZ insert starting from aa 923 to the carboxylic terminal aa 1682 (RIZ isoform 2) and bands with a lower mass. Similarly, Western blotting analysis of nuclear fraction revealed this band pattern that increased in estradiol time course MCF-7 treatment (11). To overcome these challenges, there is the need to discover peculiar features of RIZ2 and develop new methods for its identification. In the present study, we explored for the first time the role of RIZ2 by its forced expression in HEK-293 cells, which express equal amount of RIZ1 and RIZ2 proteins. Interestingly, this ectopic expression was able to increase cell viability and had a growth-prompting effect. Additionally, the forced expression of RIZ2 caused the deregulation of several genes involved in mitosis, including the overexpression of genes coding for Cyclin B and for some subunits of the Anaphase-Promoting Complex/Cyclosome (APC/C), which are often dysregulated during tumorigenesis (67). Accordingly, cell cycle analysis showed that RIZ2 could facilitate the G2-to-M phase transition. Altogether, these findings suggest that the imbalance in the amount of *PRDM2* products in favor of RIZ2 modifies the expression pattern of PRDM2 target genes involved in the promotion of cell division. Moreover, our data indicated also that RIZ2 over-expression induced the increase in the size of organoids. Organoid culture is largely accepted to study stem cells, organ development and patient-specific diseases (62). Noteworthy, the role of RIZ2 on organoid growth has not been reported so far. Additionally, in our study, we set up, for the first time, specific conditions to perform organoid culture of HEK 293 cells. We found that organoids were formed after 3-days of culture in Matrigel and that RIZ2 overexpression significantly enhanced their growth already after 10 days of culture if compared with pEGFP HEK293-derived organoids, used as controls. Provided that 3D models recapitulate more reliably the features of human cancers, these data address useful information about the role of RIZ2 in tumorigenesis that might be transferred to clinical practice and drug screening. In these cells, RIZ2 increase was also accompanied by a concomitant decrease of RIZ1 transcription. Thus, RIZ2 could exert also its oncogenic functions by impairing other mechanisms involving the methyltransferase activity of RIZ1, such as DSB repair (3, 28).

Our previous investigation demonstrated that RIZ1 is able to negatively control breast cancer cell proliferation, confirming its role as tumor suppressor gene. In addition, it is well known that in estrogen-responsive cells and target tissues, estradiol is able to modulate the expression of RIZ isoforms by inducing a change in the balance of their intracellular concentrations (38). Indeed, selective RIZ1 silencing increased the number of MCF-7 cells undergoing cell division with an effect on the growth rate similar to the estradiol outcome (39). Therefore, this set of experiments did not reveal if the observed effect on cell proliferation was merely due to RIZ1 silencing or to the imbalance in favor of RIZ2. Here, the RIZtot silencing in MCF-7 cells and the consequent reduction of RIZ2/RIZ1 ratio determined a reduction of cell proliferation and an increase of cells number undergoing apoptosis, suggesting that the previously observed behavior was related to the imbalance in favor of RIZ2. MCF-7 cells are estrogen-responsive and express both RIZ1 and RIZ2. Moreover, RIZ2 silencing produced similar effects also in MDA-MB-231 cells, which express RIZ1 at very low levels and are unresponsive to estradiol. In this context, we observed a sharp decrease of cell number, with a concomitant change in cell cycle distribution.

Overall, these findings add new insights to the understanding of the putative mechanism of the tumor-promoting function of RIZ2. The presented results indicate that RIZ2 exerts a tumor-promoting function, most likely through the transcriptional regulation of genes involved in cell cycle progression. Although several candidate genes have been extrapolated by our microarray studies, the detailed genes still need to be confirmed by further analyses on stable clones. Our results strongly suggest that RIZ2 is a promising candidate oncogene in cancer development; however, additional attempts to discover cell partners interacting with RIZ2 are warranted to elucidate how the deregulation of RIZ2 prompts cell growth, survival, and organoid formation. Indeed, most of these proteins are still undefined. Progresses in mass spectrometry instrumentation and computational tools allow the identification of high-confidence interaction proteomes of numerous biologically relevant protein groups refining our knowledge of protein interaction networks and functions (68). In this context, a whole transcriptome analysis through next-generation sequencing technologies, accompanied by proteomic studies may be useful to define all the RIZ2 target genes, thus obtaining a landscape of genes and pathways involved in its tumor-promoting action (69). Thus, the study of transcriptome and RIZ2 interaction proteome could represent a crucial step to elucidate molecular bases of its function. Besides, the integration of genomic and proteomic analyses could be relevant to solve additional open questions in this topic. As mentioned, an altered ratio of RIZ2/RIZ1 isoforms could be determined also through DNA methylation of RIZ1 promoter CpG island; in this scenario, we could hypothesize that this methylation event might interfere with the binding of CCTC-binding factor (CTCF). The functions of this transcription factor are related to the recognition and binding of a preferentially unmethylated CpG-rich consensus sequence within several genomic sites, with a strong correlation between its global occupancy and DNA methylation (68, 70, 71). Although previous studies showed that CTCF binding could regulate DNA methylation, recently it has been indicated that also DNA methylation can direct CTCF binding (72, 73). Interestingly, chromatin immunoprecipitation sequencing (ChIP-seq) data suggested that CTCF might bind also the PRDM2 promoter (70). Thus, this method could be applied also to determine the degree to which CTCF occupancy could be actively inhibited *via* DNA methylation. Finally, CTCF could also function as interaction partner of RIZ1 and/or RIZ2 proteins, as previously demonstrated for Prdm5 in mouse embryonic stem cells by high-throughput technologies (74).

Although this study is still preliminary and no clinical-derived samples were analyzed, our results could have a clinical significance. Indeed, we have shown that RIZ2 overexpression caused the deregulation of several genes involved in mitosis. Significantly, many of these genes are deregulated in cancer and/or have been suggested as potential cancer therapeutic targets (67). For instance, based on the Cancer Genome Atlas data, *CCNB2* is upregulated in advanced tumor stage and correlates with poor prognosis in breast cancer (75); also, *PSMD10* is upregulated in various cancers and the use of agents directed against its protein product gankyrin has been recently indicated as a promising cancer therapeutic and preventive strategy (76). Likewise, aberrant increases of APC10 and APC11 proteins, which are two subunits of the APC/C encoded by *ANAPC10* and *ANAPC11* genes, have been recently evidenced in non-small cell lung and colorectal cancer tissues (77, 78); interestingly, inhibitors of APC/C activity are currently under investigation (67).

In summary, this study provides evidence that RIZ2 overexpression fosters viability, cell cycle progression, and organoid formation. Taken together, our findings indicate that RIZ2 upregulation may contribute to tumor promotion, supporting the hypothetical oncogenic role of this PRDM2 protein isoform. Further investigations are required to explore the RIZ2 molecular mechanisms of action in cancer and discover cell partners interacting with RIZ2. Understanding the RIZ2 functions and interacting partners would provide new hints in the discovery of diagnostic and therapeutic strategies in human cancers. Obviously, studies analyzing the overexpression of RIZ2 isoform in tumor specimens are needed; furthermore, integrated analysis would be also useful to elucidate its impact on the deregulation of genes involved in mitosis.

## Data Availability Statement 

The datasets presented in this study can be found in online repositories. The names of the repository/repositories and accession number(s) can be found below: https://www.ncbi.nlm.nih.gov/geo/, GSE150031.

## Author Contributions

CA, PG, MR, EDZ, and AC conceived the study. AS, CR, MMM, EDZ, MDD, VC, and MR performed the experiments. MR, MDD, VC, EDZ, PG, AC, and CA analyzed the data. AS, MMM, and MR performed bioinformatics analyses. MR, EDZ, MDD, and VC prepared the figures. EDZ, CA, MR, and AC wrote the original manuscript draft. LA, GC, PG, AC, and CA reviewed and edited the paper. All authors contributed to the article and approved the submitted version.

## Funding

This work was supported by University of Campania “Luigi Vanvitelli,” VALERE program to GC (ID 263), CA, and VC (ID 342); Italian Ministry of University and Scientific Research (P.R.I.N. 2017EKMFTN_002) to GC. MDD was supported by a fellowship of ‘Fondazione Umberto Veronesi’ (FUV Post-doctoral fellowship 2020, until 09/2020) and iCURE Project (B21C17000030007- Regione Campania) from 10/2020.

## Conflict of Interest

The authors declare that the research was conducted in the absence of any commercial or financial relationships that could be construed as a potential conflict of interest.
