# Supplementary material for: Searching for a Putative Mechanism of RIZ2 Tumor-Promoting Function in Cancer Models
Source: Front Oncol. 2021 Jan 29;10:583533. doi: 10.3389/fonc.2020.583533 (PMC7880127; doi:10.3389/fonc.2020.583533)
Supplement: Supplementary file 1 [file DataSheet_1.docx]

Supplementary Material


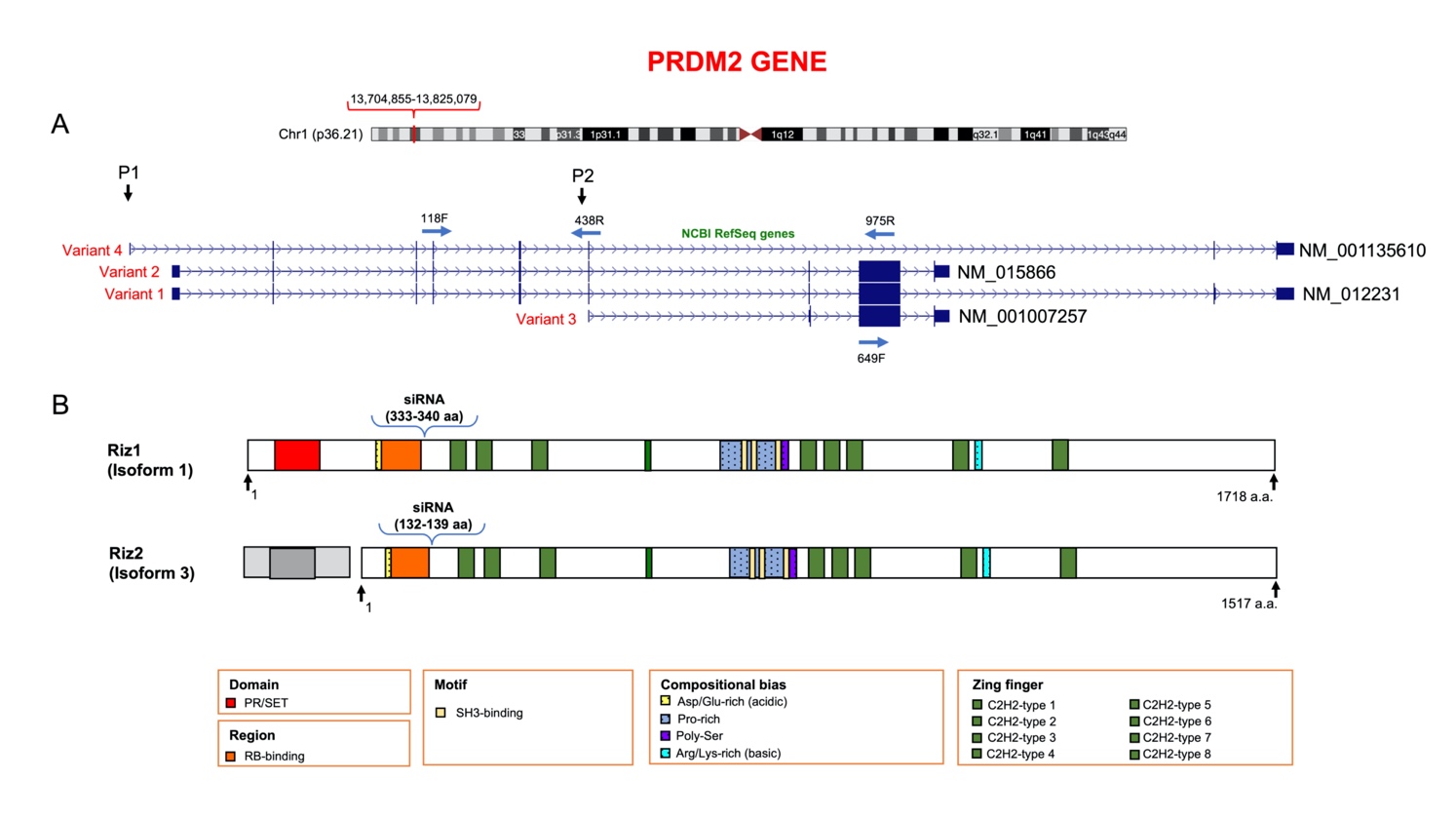


Supplementary Figure S1. PRDM2 Structure. A) Graphic illustration of human *PRDM2* gene with its variants and schematic localization of primers utilized to evaluate the expression levels of RIZ1 and RIZtot. P1 and P2 represent the positions of the different promoters. B) Structure of human RIZ1 and RIZ2 proteins, as annotated in the UniProt database. Conserved residues/motifs of the PR domain are illustrated in the lower section of the figure. The position of utilized siRNAs on target aa residues is also depicted.

**Supplementary Figure S2**. SP-PIR keywords of enriched DEGs. The top SP-PIR keywords of enriched enrichment of differentially expressed genes (DEGs) are showed. DEGs were related to cytoplasm, acetylation, nucleus, alternative splicing, phophoprotein.

**Supplementary Figure S3**. Study of cell cycle regulation. (A) Histogram Plots of cell cycle distribution. Upper panel: FACS analysis performed after 18 hours of nocodazole treatment; bottom panel: FACS analysis performed after 3 hours of release from nocodazole treatment; (B) cell cycle analysis. Graphs show the mean of at least three independent experiments with error bars indicating standard deviation. Values are mean ± standard deviation (SD) of biological triplicates. ****p-value ≤ 0.0001, ***p-value ≤ 0.001, **p-value ≤ 0.01, *p-value ≤ 0.05, ns p-value > 0.05 vs. control cells.


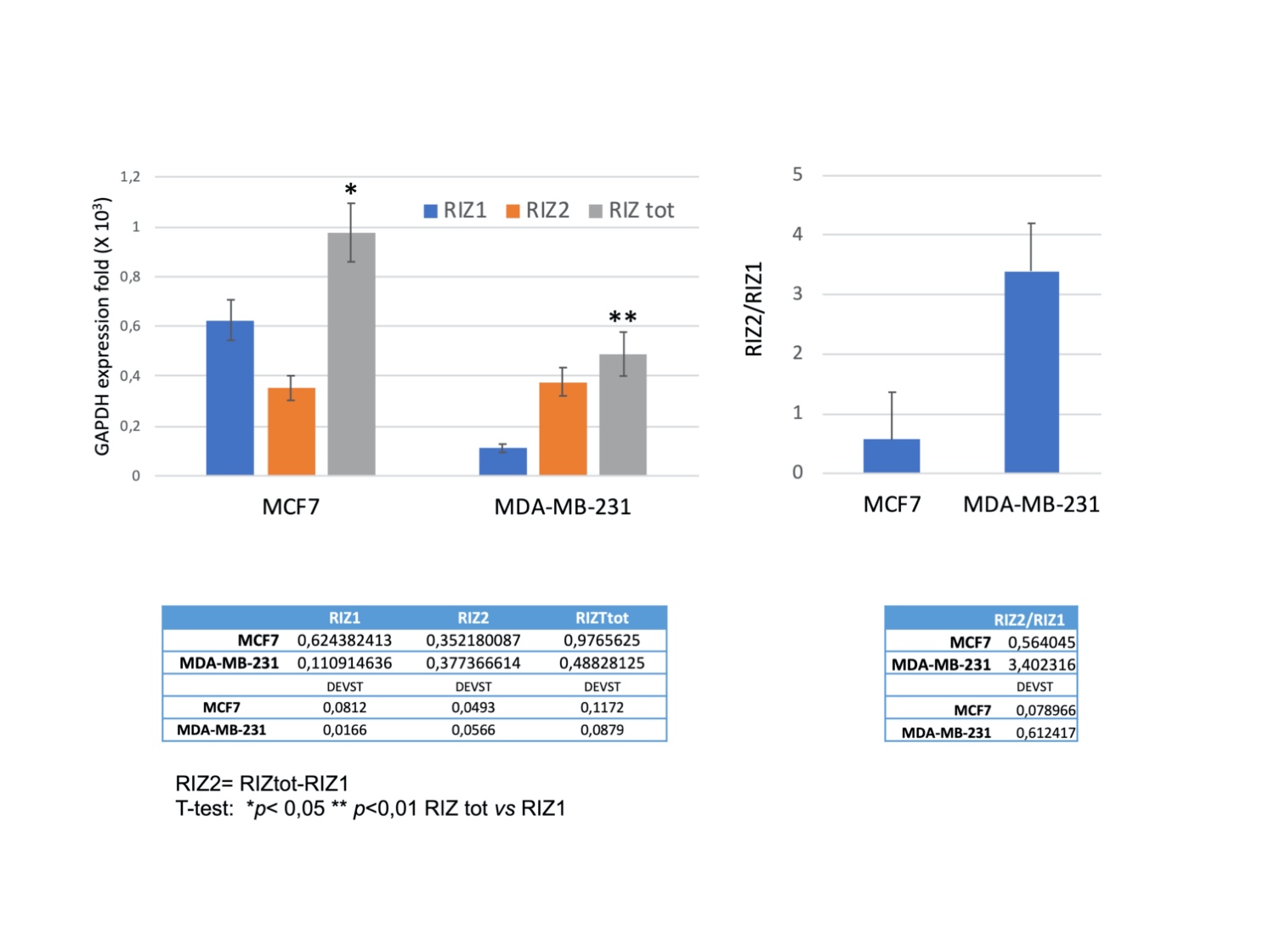


**Supplementary Figure S4**. Expression level of RIZ1 and RIZ2 transcripts and RIZ2/RIZ1 ratio in MCF-7 and MDA-MB-231 cells.

**Supplementary Table S1:** Biological process categories.

| GO Term | Description | Count | ***p*-**Value |
| --- | --- | --- | --- |
| **Up-regulated** | | | |
| GO:0065003 | macromolecular complex assembly | 22 | 6.1E-4 |
| GO:0007049 | cell cycle | 20 | 1.6E-2 |
| GO:0006396 | RNA processing | 19 | 9.5E-4 |
| GO:0010605 | negative regulation of macromolecule metabolic process | 19 | 1.9E-2 |
| GO:0044265 | cellular macromolecule catabolic process | 18 | 3.2E-2 |
| GO:0006461 | protein complex assembly | 17 | 2.6E-3 |
| GO:0022402 | cell cycle process | 17 | 7.7E-3 |
| GO:0030163 | protein catabolic process | 16 | 3.5E-2 |
| GO:0034622 | cellular macromolecular complex assembly | 15 | 2.1E-4 |
| GO:0006397 | mRNA processing | 13 | 2.4E-2 |
| GO:0032268 | regulation of cellular protein metabolic process | 13 | 4.1E-2 |
| GO:0008380 | RNA splicing | 12 | 2.7E-3 |
| GO:0022403 | cell cycle phase | 12 | 3.7E-2 |
| GO:0043065 | positive regulation of apoptosis | 12 | 4.7E-2 |
| GO:0043068 | positive regulation of programmed cell death | 12 | 4.9E-2 |
| GO:0010942 | positive regulation of cell death | 12 | 5.0E-2 |
| GO:0006412 | translation | 11 | 2.1E-2 |
| GO:0000278 | mitotic cell cycle | 11 | 4.2E-2 |
| GO:0048534 | hemopoietic or lymphoid organ development | 10 | 1.3E-2 |
| GO:0002520 | immune system development | 10 | 1.8E-2 |
| **Down-regulated** | | | |
| GO:0043933 | macromolecular complex subunit organization | 20 | 4.9E-3 |
| GO:0009057 | macromolecule catabolic process | 19 | 2.5E-2 |
| GO:0046907 | intracellular transport | 18 | 1.0E-2 |
| GO:0065003 | macromolecular complex assembly | 18 | 1.1E-2 |
| GO:0044265 | cellular macromolecule catabolic process | 17 | 4.7E-2 |
| GO:0006396 | RNA processing | 16 | 9.8E-3 |
| GO:0070271 | protein complex biogenesis | 14 | 2.5E-2 |
| GO:0006461 | protein complex assembly | 14 | 2.5E-2 |
| GO:0006412 | translation | 12 | 6.9E-3 |
| GO:0000278 | mitotic cell cycle | 12 | 1.4 E-2 |
| GO:0016071 | mRNA metabolic process | 12 | 1.4 E-2 |
| GO:0008380 | RNA splicing | 11 | 6.6E-3 |
| GO:0034621 | cellular macromolecular complex subunit organization | 11 | 2.8E-2 |
| GO:0006091 | generation of precursor metabolites and energy | 10 | 3.1E-2 |
| GO:0006397 | mRNA processing | 10 | 3.6E-2 |

**Supplementary Table S2:** Cellular component categories.

| Term | Description | Count | *p-*Value |
| --- | --- | --- | --- |
| **Up-regulated** | | | |
| GO:0043232 | intracellular non-membrane-bounded organelle | 58 | 2.7E-04 |
| GO:0031974 | membrane-enclosed lumen | 55 | 1.0E-07 |
| GO:0070013 | intracellular organelle lumen | 52 | 4.2E-07 |
| GO:0043233 | organelle lumen | 52 | 8.5E-07 |
| GO:0031981 | nuclear lumen | 45 | 7.3E-07 |
| GO:0005739 | mitochondrion | 35 | 9.4E-06 |
| GO:0005829 | cytosol | 34 | 1.0 E-03 |
| GO:0005654 | nucleoplasm | 32 | 2.3E-06 |
| GO:0031090 | organelle membrane | 27 | 6.7E-03 |
| GO:0044429 | mitochondrial part | 26 | 1.1E-06 |
| GO:0031967 | organelle envelope | 23 | 7.0E-05 |
| GO:0031975 | envelope | 23 | 7.4E-05 |
| GO:0005740 | mitochondrial envelope | 20 | 8.3E-06 |
| GO:0031966 | mitochondrial membrane | 18 | 4.7E-05 |
| GO:0030529 | ribonucleoprotein complex | 18 | 1.0 E-03 |
| GO:0044451 | nucleoplasm part | 18 | 2.4 E-03 |
| GO:0005730 | nucleolus | 17 | 4.0 E-02 |
| GO:0019866 | organelle inner membrane | 16 | 7.2E-05 |
| GO:0015630 | microtubule cytoskeleton | 16 | 1.1 E-02 |
| GO:0005743 | mitochondrial inner membrane | 15 | 1.2E-04 |
| GO:0005694 | chromosome | 13 | 3.1 E-02 |
| GO:0044455 | mitochondrial membrane part | 11 | 1.1E-05 |
| **Down-regulated** | | | |
| GO:0043232 | intracellular non-membrane-bounded organelle | 52 | 1.8E-03 |
| GO:0043228 | non-membrane-bounded organelle | 52 | 1.8E-03 |
| GO:0031974 | membrane-enclosed lumen | 46 | 3.5E-05 |
| GO:0070013 | intracellular organelle lumen | 45 | 2.7E-05 |
| GO:0043233 | organelle lumen | 45 | 4.8E-05 |
| GO:0031981 | nuclear lumen | 38 | 7.49E-05 |
| GO:0031090 | organelle membrane | 31 | 1.2E-04 |
| GO:0005739 | mitochondrion | 27 | 2.5-03 |
| GO:0031967 | organelle envelope | 22 | 8.1E-05 |
| GO:0031975 | envelope | 22 | 8.5E-05 |
| GO:0005783 | endoplasmic reticulum | 22 | 1.7-02 |
| GO:0005730 | nucleolus | 21 | 1.0-03 |
| GO:0005654 | nucleoplasm | 20 | 2.6-02 |
| GO:0044429 | mitochondrial part | 19 | 1.0-03 |
| GO:0019866 | organelle inner membrane | 16 | 3.5E-05 |
| GO:0031966 | mitochondrial membrane | 16 | 2.6E-04 |

**Supplementary Table S3:** Molecular function categories.

| Term | Description | Count | *p-*Value |
| --- | --- | --- | --- |
| **Up-regulated** | | | |
| GO:0003723 | RNA binding | 28 | 2.0E-06 |
| GO:0005198 | structural molecule activity | 16 | 3.0E-02 |
| GO:0003712 | transcription cofactor activity | 11 | 2.9E-02 |
| **Down-regulated** | | | |
| GO:0000166 | nucleotide binding | 47 | 3.1E-03 |
| GO:0017076 | purine nucleotide binding | 36 | 5.0E-02 |
| GO:0003723 | RNA binding | 22 | 9.6E-04 |

**Supplementary Table S4:** Kegg pathway analysis

| Pathway | Gene Count | p-Value | Symbol Gene |
| --- | --- | --- | --- |
| **UP-regulated DEGs** | | | |
| Oxidative phosphorylation | 9 | 5.9E-4 | ATP5G1, ATP5G3, ATP5L, COX17, COX2, COX3, NDUFC1, NDUFC2, COX4I1 |
| Cell cycle | 8 | 2.3E-3 | ANAPC10P1, ANAPC11, ANAPAC1, ANAPAC4, Bub3, CCNB2, CDKN2C, TFDP2 |
| **Down-regulated DEGs** | | | |
| Spliceosome | 7 | 1.1E-2 | PRPF31, RBM22, THOC2, EFTUD2, PPIE, SNRNP200, SFRS9, |
